# Interaction of chromatin with a histone H1 containing swapped N- and C-terminal domains

**DOI:** 10.1042/BSR20150087

**Published:** 2015-06-22

**Authors:** Jordana B. Hutchinson, Manjinder S. Cheema, Jason Wang, Krystal Missiaen, Ron Finn, Rodrigo Gonzalez Romero, John P. H. Th’ng, Michael Hendzel, Juan Ausió

**Affiliations:** * Department of Biochemistry and Microbiology, University of Victoria, Victoria, British Columbia, Canada V8W 3P6; † Department of Oncology, Faculty of Dentistry and Medicine, University of Alberta, Edmonton, Alberta, Canada T6G 1Z2; ‡ Northern Ontario School of Medicine, 955 Oliver Road, Thunder Bay, Ontario, Canada P7B 5E1

**Keywords:** analytical ultracentrifuge, chromatin, circular dichroism, FRAP, histones

## Abstract

Although the details of the structural involvement of histone H1 in the organization of the nucleosome are quite well understood, the sequential events involved in the recognition of its binding site are not as well known. We have used a recombinant human histone H1 (H1.1) in which the N- and C-terminal domains (NTD/CTD) have been swapped and we have reconstituted it on to a 208-bp nucleosome. We have shown that the swapped version of the protein is still able to bind to nucleosomes through its structurally folded wing helix domain (WHD); however, analytical ultracentrifuge analysis demonstrates its ability to properly fold the chromatin fibre is impaired. Furthermore, FRAP analysis shows that the highly dynamic binding association of histone H1 with the chromatin fibre is altered, with a severely decreased half time of residence. All of this suggests that proper binding of histone H1 to chromatin is determined by the simultaneous and synergistic binding of its WHD–CTD to the nucleosome.

## Abstract (precis)

The present study was to understand whether the globular or C-terminal linker histone domain is more important for its binding to chromatin. Using histone H1, with swapped domain orientation, we found that both domains are equally important for nucleosome binding.

## INTRODUCTION

The chromatin fibre is a highly dynamic nucleoprotein complex whose major constituents are the genomic DNA and histones. Histones are rather small basic proteins that can be structurally and functionally grouped into two major distinct families: core histones, consisting of a central core histone fold [1] domain (CHFD) [2] which is flanked by intrinsically disordered N- and C- terminal tails and linker histones or histones of the H1 family, which consist of a winged helix domain (WHD) [3–5] that is also flanked by intrinsically disordered N- and C-terminal tails. The core histones form the nucleosome ‘core’ around which the DNA wraps in approximately one-and-three-quarter left-handed super-helical turns, comprising approximately 145–147 bp of DNA. This association results into the most elementary subunit of chromatin, known as the nucleosome core particle (NCP) [6], which was crystallized in 1997 [7]. Linker histones bind to the linker DNA regions at the entry and exit sites of the nucleosome resulting in a structure that protects the DNA from digestion by micrococcal nuclease by an additional 20–22 bp termed as chromatosome [8]. In the chromatin fibre, NCPs are connected by variable-size stretches of linker DNA (0–100 bp) that bind to ‘linker’ histones which modulate the dynamics and extent of the folding of the chromatin complex [9,10].

In comparison with core histones, linker histones have been much less studied, due in part to the higher complexity of their post-translational modifications (PTMs) [11,12] resulting from the larger number of targeting sites and the micro-heterogeneity of the histone H1 isoforms [13]. Also, the highly dynamic nature of their binding to chromatin [14] makes it difficult to perform reliable ChIP analyses even under cross-linking conditions. The same is also true at the structural level and, only until very recently, have a partial crystallographic image [15] and high resolution NMR [16] images of their association with the nucleosome been produced.

There is still an unresolved issue regarding the mechanism involved in the association of histone H1 with the linker regions flanking the nucleosome [17,18]. Although for a long time, the C-terminal domain (CTD) has been deemed critically important [19,20], it remains still unclear whether this is the primary determinant for proper binding to chromatin or whether the WHD needs to first recognize the intrinsic structural features of DNA at its entry and exit sites of the nucleosome. In the present study, we have designed a synthetic recombinant H1 with swapped N- and C-terminals that should allow us to discern between the two possibilities. Our results show that both the C-terminal tail and the WHD constitute a linker histone fold-binding domain and are necessary for proper binding and folding of the chromatin fibre.

## MATERIALS AND METHODS

### Cloning and sequencing

The DNA sequence of human histone H1.1 mutant with the N- and C-termini swapped was designed to encode the protein sequence shown in Figure 1(A), using a template for the DNA sequence from the cDNA sequence of the native form. The mutant sequence thus obtained was synthesized by integrated DNA technologies (IDT) and inserted in a pIDTsmart plasmid. The sequence was PCR amplified using primers containing Nde1 and BamH1 restriction sites and sub-cloned into a pET11a plasmid.

**Figure 1:**
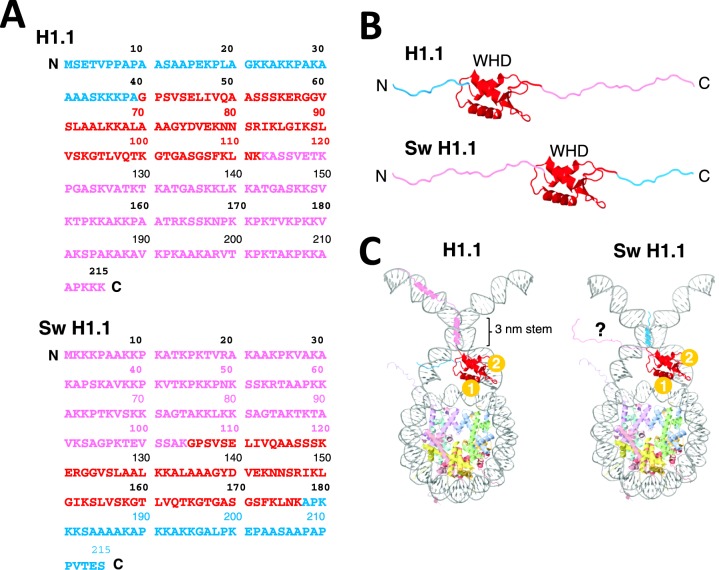
Structure of wild type and swapped histone H1.1 (**A**) Amino acid sequence analysis of human histone H1.1 in its native and N- and C-terminal swapped conformation. (**B**) Schematic representation of the tertiary structure organization of human histone H1.1. (**C**) Schematic representation of the nucleosome organization upon binding of histone H1. In this organization, the CTD of the native H1 form adopts an α-helical conformation upon binding to DNA [58] in the nucleosome [59]. The 3-nm stem resulting from the interaction of this region with the linker DNA is also indicated [16,34,60]. The S1 and S2 sites of interaction of the WHD with the nucleosomal DNA [35] are shown in orange. In all these representations, the regions corresponding to the N-terminal, WHD and the CTD in the native form are represented in blue, red and pink respectively.

### Recombinant protein expression and purification

The swapped H1.1 and the native version of H1.1, both in pET11a, were expressed in *Escherichia coli* BL21 cells. The cellular pellets thus obtained were resuspended in a mixture of 6 M guanidinium HCl, 1 mM EDTA, 1 mM DTT and 50 mM Tris/HCl (pH of 7.5). The cell mixture was then dounce homogenized with 40 strokes on ice. This mixture was dialysed at 4°C in 0.1 M NaCl, 50 mM Tris/HCl (pH 7.5) and 1 mM EDTA for 2 h. One volume of 10% perchloric acid (PCA) was added to the mixture and this mixture was centrifuged at 27000 ***g*** for 15 min at 4°C. The supernatant was then brought to 0.2 N HCl by addition of 1/60 volume of 12 N HCl and proteins in the extract were precipitated with six volumes of acetone after overnight incubation at −20°C. The acetone protein precipitate was recovered by centrifugation at 12000 ***g*** at 4°C for 10 min. The pellet was resuspended in an equal volume of fresh acetone at room temperature and centrifuged again. This step was repeated twice and the pellet was finally dried under vacuum. The dried protein extract was dissolved in water and reversed phase HPLC-purified as described elsewhere [21].

### MS

The molecular mass of the swapped and native histone H1.1 were analysed by MALDI-TOF; MS using a sinapinic acid matrix dissolved in 50% acetonitrile, 50% water and 0.1% TFA (trifluoroacetic acid).

### Histone renaturation

Both the native and swapped histone H1.1 were re-natured after HPLC [21]. To this end, the protein solutions were lyophilized overnight and then dissolved in 6 M guanidinium hydrochloride, 20 mM β-mercaptoethanol and 50 mM Tris/HCl (pH 7.5). This solution was incubated at room temperature for 30 min; sterile distilled water was then added. The protein was then dialysed for 4 h against distilled water at 4°C, followed by an overnight dialysis at 4°C against 2 M NaCl, 50 mM Tris/HCl (pH 7.5), 1 mM EDTA buffer. The samples were finally dialysed for 4 h at 4°C against 2 l of 20 mM sodium phosphate (pH 7.2) buffer.

### Gel electrophoresis

The proteins were analysed by SDS/PAGE according to [22]. Electrophoretic analysis of DNA and nucleosome complexes was carried out in either agarose (1%) in 40 mM Tris-acetate (pH 8.0), 1 mM EDTA buffer or in native PAGE 4.5% polyacrylamide as described in [23].

### CD

CD was carried out on a Jasco-700 series spectropolarimeter, with the Spectra Manager software used to analyse the data. The spectra were taken from 260 to 190 nm, with the proteins at a concentration of 4.8 × 10^−6^ M for the swapped protein and 3.4 × 10^−6^ M for the wild-type H1.1 in 20 mM sodium phosphate (pH 7.2) buffer. A native H1 mixture isolated from HeLa cells was used as a control. A 0.1 cm cell was used and the remaining conditions were as described in [24].

### Nucleosome reconstitution

Nucleosomes were reconstituted as described elsewhere [25] using a 208-bp DNA fragment and chicken erythrocyte core histones that had been purified by hydroxylapatite chromatography [26]. The 208-bp fragment was obtained by RsaI digestion of a 208–212 DNA construct consisting of 12 identical copies of a 196-bp nucleosome-positioning fragment obtained from the 5S rRNA gene of the sea urchin *Lytechinus variegatus* [27]. The RsaI digestion produces a fragment consisting of approximately 20 and 40 nts at each side of the 146-bp positioning sequence.

### EMSA

Titration of nucleosomes with histone H1 was carried out by EMSA using 0.9% agarose gels in 45 mM Tris base, 45 mM boric acid, 1.25 mM EDTA [0.5× TBE (Tris-borate–EDTA)] buffer and following the protocol described in [28].

### Linker histone depletion of chromatin and histone H1 re-addition

Long chromatin was obtained from [MDV (Marek's disease virus)-transformed spleen lymphoma induced by the BC strain] (MSB) chicken cells, as described in [29]. After linker histone depletion [29], the linker depleted chromatin at approximately 50 μg/ml was dialysed against 0.5 M NaCl 10 mM Tris/HCl (pH 7.5) 0.25 mM EDTA at 4°C and mixed with either recombinant histone H1.1 or its swapped version in the same buffer. The mixtures were carried out at 1 or 1.5 mol of histone H1 per 1 mol of nucleosome equivalent and dialysed extensively against 0.3 M NaCl 10 mM Tris/HCl (pH 7.5) 0.25 mM EDTA4°C. This ratio corresponds approximately to a nominal w/w ratio of histone H1:nucleosome equivalent of 1:10, considering the molecular mass of histone H1 (22000–23000) and that of a 180–190 bp nucleosome (approximately 230000) The samples were finally dialysed overnight at 4°C against either 5 mM NaCl, 10 mM Tris/HCl (pH 7.5), 0.1 mM EDTA or 80 mM NaCl, in the same buffer. The concentration of chromatin was approximately 30–40 μg/ml.

### HeLa native H1 preparation

Linker histones in their native conformation were obtained from HeLa cell chromatin as described elsewhere [30].

### Analytical ultracentrifuge analysis

Analytical ultracentrifuge analysis was carried out on a Beckman XL-I ultracentrifuge using an An-55 aluminium rotor and double sector aluminium-filled Epon centrepieces. Sedimentation velocity runs were conducted at 20°C at a speed of 16000 rpm. Plots were generated using Ultrascan II software using van Holde-Weischet analysis [31].

### FRAP

FRAP analyses were performed using N- and C-terminally GFP-tagged versions of the native and swapped histone H1 [32] as described elsewhere [33].

## RESULTS

### A synthetic histone H1.1 with swapped N- and C-terminal domains

We designed a synthetic recombinant histone H1.1 with swapped N-terminal domain (NTD) and CTD, henceforth as ‘swapped H1.1’. This organization is formed by flipping the original NTD of H1.1 and attaching its C terminal end to the C-terminal end of WHD and flipping the CTD of the histone and attaching its NTD to the N-terminal end of WHD (Figures 1A and 1B). Figure 1(C) shows a schematic representation of the H1-containing nucleosome organization, based on the NMR structure [16] and single base pair resolution mapping [34] and the anchoring S1 and S2 sites of the WHD to the nucleosome [16,35]. The N-/C-terminal swapping performed by us changes the orientation of the WHD with regards to the CTD and NTD and thus provides a unique tool to understand the importance of the role of the WHD in the proper binding of linker histones to the chromatin fibre.

A cDNA sequence encoding the swapped version of histone H1.1 was synthesized using the codon usage of the native form and was bacterially expressed and purified as shown in the inset of Figure 2. The proteins were then re-natured using a previously described protocol from our laboratory [21,25] and their secondary structure organization was determined by CD (Figure 2).

**Figure 2:**
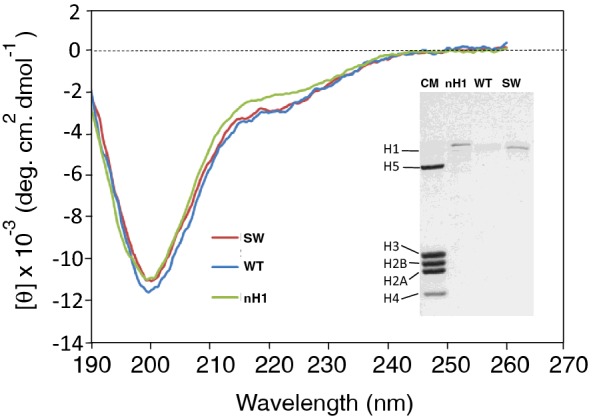
CD spectra of histone H1.1 CD spectra of the recombinant wild-type histone H1.1 (WT) in comparison with the recombinant H1.1 consisting of swapped NTD and CTD (SW) and in comparison with a native histone H1 mixture (nH1) obtained from HeLa cells. The insert shows an SDS/PAGE of the samples used in this analysis.

The CD spectra of the swapped and native histone H1.1 versions were compared with the spectrum obtained from a native HeLa histone H1 sample consisting of a mixture of the different histone micro-heterogeneous variants which are characteristic of this histone family [13]

The CD spectra thus obtained shows a negative ellipticity peak at 200 nm, which is the characteristic peak of a random coil organization and a broad negative shoulder at 220–222 nm representing the α-helical contribution [36], as would be expected from the histone H1 conformation; it is possible to use the ellipticity at 220 nm to estimate the α-helical content of the protein [36]. With this, we estimated that the reconstituted native forms of H1.1 and swapped H1.1 were approximately 17% for both of them, as their spectra in that region are completely overlapping. This value is very close to the value of 19.5% estimated for the erythrocyte-specific linker histone H5 from chickens, calculated from the crystallographic analysis of its WHD and assuming a random coil conformation for the rest of the molecule in solution [37]. In addition, the spectra of the recombinantly expressed and reconstituted wild-type and swapped form of histone H1.1 are very similar to the spectrum for the native mixture of histone H1 from Hela cells. The slightly lower ellipticity at 220 nm in this case might simply reflect the micro-heterogeneous [13] nature of this mixture, which consists of at least of five different variants.

### Native and swapped H1.1 can recognize the architectural features of nucleosome-organized chromatin

The two forms of recombinant wild-type and swapped histone H1.1 were next used to reconstitute long chromatin fragments that had been stripped of their native linker histones. A time-course micrococcal nuclease digestion was then performed. The digestion was stopped by addition of SDS to 0.3% and the samples were loaded on a native PAGE; the results are shown in Figure 3(A). Quite unexpectedly, the chromatin sample that had been reconstituted with the swapped H1.1 version exhibited a micrococcal-nuclease resistant of an apparent larger size than that observed for chromatin, which had been reconstituted with the wild-type version of histone H1.1 (Figure 3A).

**Figure 3:**
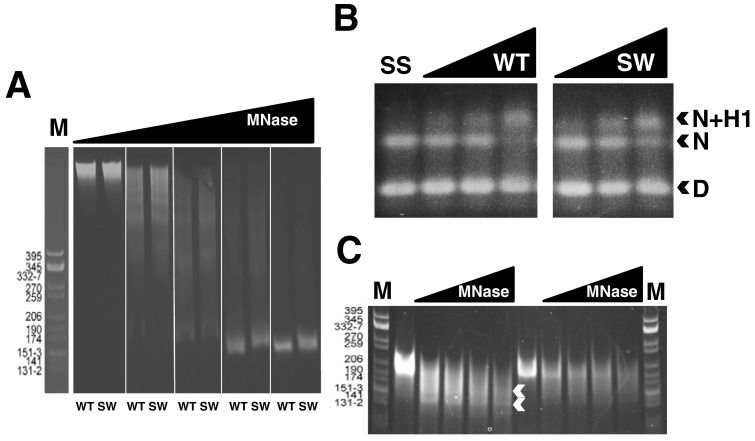
Binding of histone H1.1 to chromatin (**A**) Native (4%) PAGE of a micrococcal nuclease digestion of histone H1-depleted chromatin reconstituted with recombinant H1.1 (WT) or H1.1 consisting of swapped NTD and CTD (SW). (**B**) Agarose (0.9%) gel electrophoresis of 208-bp reconstituted nucleosomes upon further reconstitution with increasing amounts (120, 240 and 480 ng) of either recombinant H1.1 (WT) or H1.1 consisting of swapped NTD and CTD (SW). (**C**) Micrococcal nuclease (Mnase) digestion of the samples shown in (**B**). The two white arrows point to the 168-bp (upper) and 145-bp (lower) Mnase resistant bands. (**D**) free, 208-bp DNA; N, nucleosome; M is a CfoI-digested pBr322 plasmid DNA used as a marker. The base pair numbers corresponding to the different bands are indicated in the left-hand side of the gels. SS, starting sample.

In an attempt to figure out the answer to this surprising result, we reconstituted Simpson's 5S-208 bp nucleosomes [27] with both versions of the histone H1.1. The experiments were carried out under the same identical conditions described in [28]. The results of the reconstitution are shown in Figure 3(B). As it can be seen, both H1.1 and its swapped version preferentially bind to the nucleosomally arranged DNA to produce a distinct band [(N+H1) Figure 3B], which corresponds to a chromatin particle consisting of a nucleosome and a molecule of linker histone [28]. The swapped version exhibits a slightly lower efficiency, as still some unbound nucleosomes can be observed (lane 7 in Figure 3B) at an equivalent protein loading for which almost all the nucleosomes are shifted with the wild-type form (lane 4, Figure 3B). The particle corresponding to the maximum shift was then digested with increasing amounts of micrococcal nuclease (Figure 3C). The results of such digestion show that whereas the particle reconstituted with wild-type is digested down to a 168 and 145 bp as expected, the particle reconstituted with swapped H1.1 is more resilient to digestion. The 168-bp band and 145–147 bp correspond to the micrococcal nuclease pauses of digestion at the chromatosome and the NCP boundaries respectively.

All this indicates that both the wild-type and the swapped form of H1.1 are able to preferentially recognize and bind to the four-way junction-like DNA structure [38–40] at the entry and exit sites of the DNA in the nucleosome. This feature is probably the result of both proteins sharing an identical WHD [38,40].

### Swapped histone H1.1 is unable to fold chromatin properly and exhibits a faster dissociation rate

The histone H1.1 reconstituted chromatin samples described in the previous section were then analysed at low (5 mM NaCl) and high ionic strength (80 mM NaCl) by analytical ultracentrifuge analysis [41,42]. The results are shown in Figure 4. Under these conditions, chromatin exhibits an unfolded and tightly folded organization respectively [43]. As a result, the sedimentation coefficient of the samples increases in a way that is dependent on the number of nucleosomes of the chromatin fragment [41,42] and the presence or the absence of histone H1 [44,45] (compare Figures 4A and 4B).

**Figure 4:**
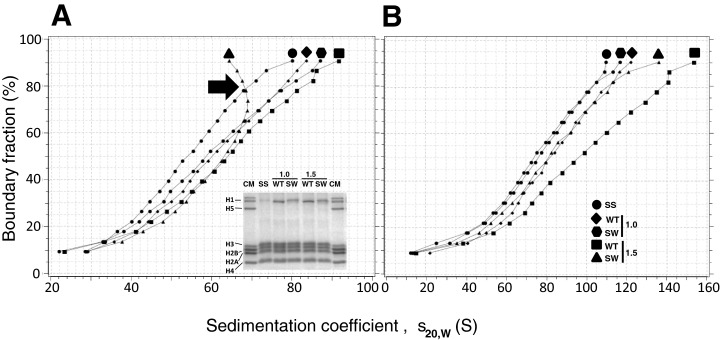
Histone H1.1-dependent chromatin folding Analytical ultracentrifuge analysis showing the integral distribution of the standard sedimentation coefficients (s_20,w_) of a histone H1-depleted chromatin fraction reconstituted with recombinant H1.1 (WT) or H1.1 consisting of swapped NTD and CTD (SW) at different ionic strength conditions: 5 mM NaCl, 10 mM Tris/HCl (pH 7.5) 0.1 mM EDTA (**A**) or 80 mM NaCl, 10 mM Tris/HCl (pH 7.5) 0.1 mM EDTA (**B**). The insert in (**A**) shows an SDS/PAGE of the histone composition of the samples used in the analysis at a ratio of 1.0 or 1.5 (mol H1, mol nucleosome).

As shown in Figure 4, all the H1.1-cotaining samples generally show an increased sedimentation coefficient when compared with the starting linker histone-stripped fraction, as is to be expected [44]. The increase in the slope of the sedimentation coefficients of the complexes fully saturated with wild-type H1.1 is approximately in the range of 1.1–1.2 at 5 mM NaCl and 1.3–1.4 at 80 mM NaCl [46] (Figures 4A and 4B ■). The ratio 1:5 mol histone H1:1 mol of nucleosome equivalent in the chromatin complex represents a saturation ratio similar to that observed in chicken erythrocyte chromatin [see CM (chicken erythrocyte histone marker) in inset to Figure 4A]. A ratio of approximately 1.3 mol linker histone:1 mole nucleosome has been reported for chicken erythrocyte chromatin [47]. Interestingly, the sample containing 1.5:1 molar ratio of swapped H1.1 per nucleosome in the chromatin complex exhibits an unusual sedimentation behaviour at 5 mM NaCl, depicted by the broad arrow in Figure 4(A). We observed this result every time we performed this reconstitution at this ratio and salt concentration. This corresponds to a fraction of the sample containing the longer chromatin complexes aggregating very quickly along the boundary and precipitating at the bottom of the ultracentrifuge cell. This aggregation can only be explained by an inter-complex cross-linking by the swapped histone H1.1. This behaviour was never observed at the 80 mM NaCl concentration (see Figure 4B). At this salt, the swapped H1.1 saturated chromatin complex folding was impaired and it behaved similarly to the unsaturated wild-type H1.1 complexes.

To gain further understanding of the problem, we performed FRAP experiments (Figure 5). This Figure shows several interesting results. Whereas the N-terminal GFP tagged form of wild-type H1.1 we obtained showed a pattern that was identical to what had been reported previously for the same construct [48], the C-terminally tagged construct exhibited a much shorter recovery time, underscoring the importance of the CTD of H1.1 for the proper binding to chromatin. The CTD is critical for the adequate formation of the chromatosome and the ensuing folding of the chromatin fibre [2]. Therefore, addition of GFP to this end of the molecule would be expected to interfere with these processes and hence with the overall binding ability of histone H1 to chromatin. Interestingly, both the N- and the C-terminally tagged constructs of the swapped H1.1 exhibited an altered chromatin-binding behaviour very similar to that exhibited by the C-terminally tagged version of the wild-type. These results being are indicative of a very weak binding by swapped H1.1.

**Figure 5:**
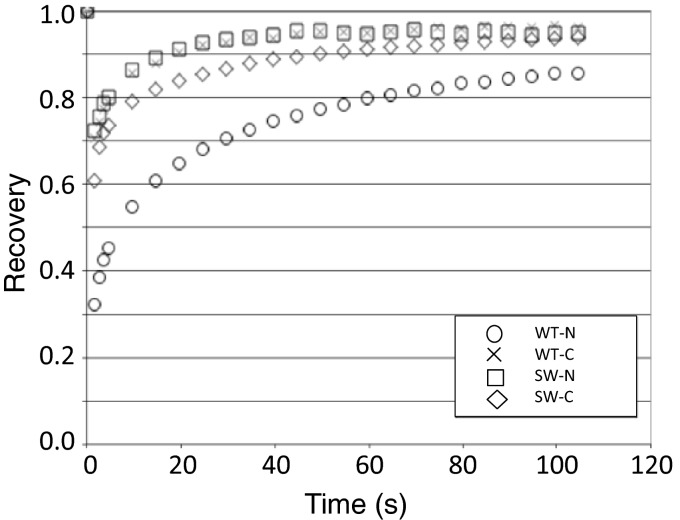
FRAP analysis of GFP-tagged H1.1 (WT) or H1.1 consisting of swapped NTD and CTD (SW) The N- and C- labels indicate N- or C-terminal location of the GFP.

## DISCUSSION

As was mentioned in the introduction, chromatin consists of two different histone types: core histones that fold the DNA into a globular structure (the nucleosome) and linker histones that bind to the linker DNA regions between adjacent nucleosomes and fold the fibre resulting from such association.

In the case of the core histones, the N- and C-terminal tails are not critical for the organization of the nucleosome. In fact, it is possible to reconstitute nucleosome particles with an almost undistinguishable conformation and stability [49], emphasizing the critical role of the histone fold domain (HFD). The tails, which were acquired later on in the process of evolution of these proteins [50], play an important role in the folding of the chromatin fibre [51,52] whereas providing a substrate for most of the epigenetically important histone PTMs. In contrast, the CTD of linker histones has been long known to be critical to its primary function in the folding of the chromatin fibre [19] and preceded the WHD early in evolution [3]. In some protists, the linker histones consist only of the CTD [53,54], compared with their most evolved forms in metazoans.

The results in the previous section, while confirming the relevance of the CTD, underscore the equally critical role played by the WHD. The results from Figure 2 clearly demonstrate that whereas both wild-type H1.1 and swapped H1.1 can recognize the four-way junction-like structure of the linker DNAs entering and exiting the nucleosome (Figure 3B), they cannot provide the proper 168-bp chromatosome organization (Figure 3C) that would be required for folding of the chromatin fibre. Furthermore, and in sharp contrast with early data [28], this indicates that the DNA-binding orientation of the WHD and hence its DNA-binding surface [35] (Figure 1C) are critical to this binding, as it clearly determines the positioning of the CTD for an adequate chromatin folding. Additional support for this conclusion comes from the analytical ultracentrifuge data shown in Figure 4 where, as it is shown in Figure 4(B), only the wild-type of H1.1 can produce the expected chromatin folding. Indeed, the aggregation results obtained for swapped H1.1 under conditions of histone H1 saturation and low salt concentration (Figure 4A arrow) suggest that the misplacing of the CTD results in a situation similar to that schematically depicted in Figure 6(B), where some of the CTD is unable to find its proper position and interacts with DNA in neighbouring chromatin fibres.

**Figure 6:**
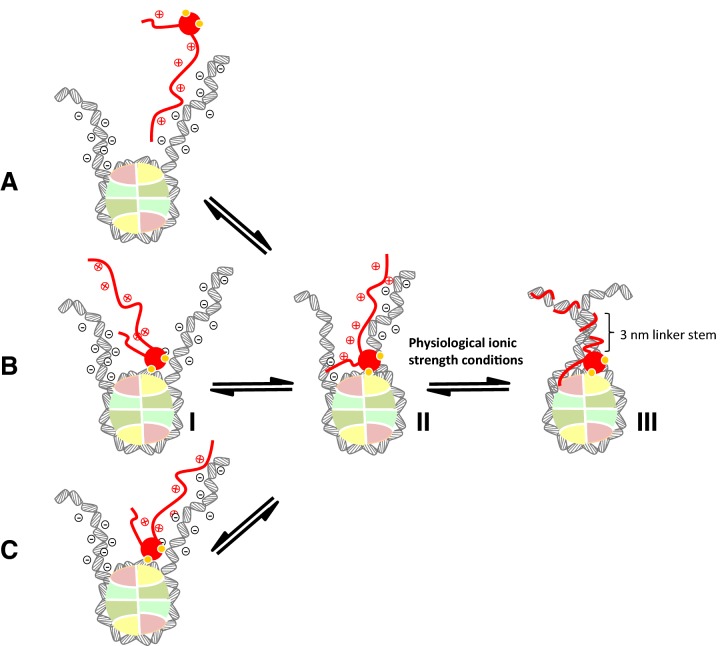
Cartoon representation of the three potential models of the association of histone H1 with the nucleosome In model (**A**), the CTD of H1 initiates the binding interacting non-specifically with the linker DNA in an electrostatically driven association. In model (**B**), the WHD, stereospecifically through its S1- and S2-binding sites (orange circles), interacts first with the linker DNA at its entry and exiting regions in the nucleosome. In model (**C**), the WHD–CTD interact simultaneously with the linker DNA region, forming an electrostatic clamp. The double arrows indicate that the association of linker histones (histone H1) is a highly reversible process, as visualized in Figure 5. Reproduced from [17]: Raghuram, N., Carrero, G., Th’ng, J. and Hendzel, M.J. (2009) Molecular dynamics of histone H1. Biochem. Cell. Biol. **87**, 189–206 with permission from Nuclear Regulatory Commission.

Not only is the swapped H1.1 version unable to properly interact with the chromatin fibre *in vitro*, but also *in vivo*, as the FRAP results unambiguously indicate (Figure 5).

The results presented in the present study are in full agreement with the recent modelling results on the dynamic condensation role of the CTD in chromatin folding [55], indicating that the asymmetry of the WHD (and hence, the proper orientation of the CTD and NTD) is responsible for the uneven interaction of the linker histones and the linker DNA. At low salt concentration, binding of histone H1 to the nucleosome produces a semi-open conformation (Figure 6 II) which results in a partial chromatin folded conformation (Figure 4A). At a higher salt concentration (Figure 6 III), the linker stem is formed yielding to a complete folding of the fibre (Figure 4B).

Moreover, our data shed light on further dynamic aspects of histone H1 binding to chromatin, related to the mode of interaction. A long-standing debate has been whether the interaction is first initiated by binding of the CTD [18] (Figure 6A), the WHD [56] (Figure 6B) or both domains at the same time (Figure 6C). All in all, the data presented in the present study support the suggested mechanism proposed earlier [17,57]. This is that the WHD–CTD in metazoans works synergistically (Figure 6C) as a linker histone fold domain (LHFD) [2] which, under physiological ionic strength conditions, is required for the recognition of the proper nucleosome binding site, simultaneously allowing for the 3-nm linker stem formation necessary for the proper folding of the chromatin fibre.

## Abbreviations

CM: chicken erythrocyte histone marker
CTD: C-terminal domain IDT, integrated DNA technologies
FRAP: fluorescence recovery after photobleaching
NCP: nucleosome core particle
NTD: N-terminal domain
PTM: post-translational modification, WHD, winged helix domain
